# Transcriptome analysis of trembling aspen (*Populus tremuloides*) under nickel stress

**DOI:** 10.1371/journal.pone.0274740

**Published:** 2022-10-13

**Authors:** Karolina M. Czajka, Kabwe Nkongolo

**Affiliations:** 1 Biomolecular Sciences Program, Laurentian University, Sudbury, Ontario, Canada; 2 Department of Biology, School of Natural Sciences, Laurentian University, Sudbury, Ontario, Canada; National Botanical Research Institute CSIR, INDIA

## Abstract

Plants have evolved heavy metal tolerance mechanisms to adapt and cope with nickel (Ni) toxicity. Decrypting whole gene expression of Trembling Aspen (*Pinus tremuloides*) under nickel stress could elucidate the nickel resistance/tolerance mechanisms. The main objectives of the present research were to 1) characterize the *P*. *tremuloides* transcriptome, and 2) compare gene expression dynamics between nickel-resistant and nickel-susceptible *P*. *tremuloides* genotypes with Whole Transcriptome (WT) sequencing. Illumina Sequencing generated 27–45 million 2X150 paired-end reads of raw data per sample. The alignment performed with StringTie Software added two groups of transcripts to the draft genome annotation. One group contained 32,677 new isoforms that match to 17,254 genes. The second group contained 17,349 novel transcripts that represent 16,157 novel genes. Overall, 52,987 genes were identified from which 36,770 genes were selected as differently expressed. With the high stringency (two-fold change, FDR value ≤ 0.05 and logFC value ≥1 (upregulated) or ≤ -1 (downregulated), after GSEA analysis and filtering for gene set size, 575 gene sets were upregulated and 146 were downregulated in nickel resistant phenotypes compared to susceptible genotypes. For biological process, genes associated with translation were significantly upregulated while signal transduction and cellular protein process genes were downregulated in resistant compared to susceptible genotypes. For molecular function, there was a significant downregulation of genes associated with DNA binding in resistant compared to susceptible lines. Significant upregulation was observed in genes located in ribosome while downregulation of genes in chloroplast and mitochondrion were preponderant in resistant genotypes compared to susceptible. Hence, from a whole transcriptome level, an upregulation in ribosomal and translation activities was identified as the main response to Ni toxicity in the resistant plants. More importantly, this study revealed that a metal transport protein (Potrs038704g29436 –ATOX1-related copper transport) was among the top upregulated genes in resistant genotypes when compared to susceptible plants. Other identified upregulated genes associated with abiotic stress include genes coding for Dirigent Protein 10, GATA transcription factor, Zinc finger protein, Auxin response factor, Bidirectional sugar transporter, and thiamine thiazole synthase.

## 1. Introduction

The effects of heavy metal contamination on plants is an important topic of research particularly for areas that have been affected by anthropogenic mining industries. In the Greater Sudbury Region (GSR), decades of nickel and copper mining activities have increased the concentrations of these heavy metals in the surrounding soil and environment [1]. Furthermore, the increased production of sulfur dioxide (SO_2_) has resulted in soil acidification which consequently makes heavy metals more bioavailable for uptake from the soil by plants [1].

Metal stress can affect the expression and activity of important antioxidant enzymes needed to deal with Reactive Oxygen Species (ROS). For instance, though nickel does not directly generate ROS because it is a redox-inactive metal [2], it has been reported to stimulate antioxidant enzymes such as superoxide dismutase (*SOD*), catalase (*CAT*), ascorbate peroxidase (*APX*), and glutathione s-transferase in plants [3–6]. The activities of these genes appeared to be reduced based on a transcriptome analysis of white birch (*Betula papyrifera*). This result is possibly due to enzyme inactivation from direct binding of Ni^2+^ to a -SH group or histidine, Ni displacement of other metals in the active binding site, or indirectly [7].

Theriault *et al*. [7] compared resistant and susceptible white birch phenotypes that were identified among a sample population treated with the same 1600 mg/kg nickel dose. Through RNA-seq analysis, significant differences in gene expression were detected among genotypes. Gene expression patterns were identified including the downregulation of genes associated with ribosomal activities and translation in resistant genotypes. However, Nkongolo *et al*. [8] detected no significant differences between nickel—resistant and susceptible red maple (*Acer rubrum*) genotypes based on transcriptome analysis with the Illumina platform.

*Populus tremuloides* is an important founder species across North America and it is a key member of the GSR ecosystem. The ability of trembling aspen to accumulate excess heavy metals in its above-ground tissues via the translocation of metals absorbed in the roots to the shoots or leaves for storage, may enable this species to better tolerate metal-contaminated-soils [9]. Numerous other characteristics of this species including high genetic variation among populations can make them hardy and adaptable to different environments [10]. This present study aims to decrypt coping mechanisms to nickel toxicity in *P*. *tremuloides* under monitored environmental conditions.

RNA-seq will help to characterize gene expression profiles of nickel-resistant and susceptible genotypes at the whole transcriptome level. This could elucidate the nickel resistance/tolerance mechanisms that may be utilized by *P*. *tremuloides* under nickel stress and to identify putative Ni transporter genes.

Hence, the specific objectives of this study are to 1) Develop and characterize the *P*. *tremuloides* transcriptome, 2) Analyze differential gene expression in nickel-resistant and susceptible *P*. *tremuloides* genotypes, and 3) Identify any novel candidate genes for nickel resistance.

## 2. Materials and methods

### 2.1. Assessment of nickel toxicity on trembling aspen (*P*. *tremuloides*) seedlings

*Populus tremuloides* seeds were collected in Woodstock, NB (seedlot# 20001001) and provided by the Canadian Forest Services seed bank in Fredericton. They were stored at 4°C until further use. Hence, no special permission was needed to collect seeds used in the present study.

Seeds were germinated in Petawawa germination boxes and seedlings were initially grown in a deep tray with soil. Four-month-old seedlings were transplanted into pots containing a 50:50 sand/soil mixture and left to grow for an additional month and a half in a growth chamber. Plants were watered as needed and fertilized twice a week with equal amounts of nitrogen, phosphorus, and potassium (20-20-20).

Nickel (Ni) toxicity was assessed by treating seedlings with an aqueous solution of nickel nitrate salt [Ni(NO_3_)_2_] at the concentrations of 1,600 mg of nickel per 1 kg of dry soil recommended in the previous studies for nickel resistance screening [9, 11]. To control for any possible effects due to the increase in nitrate ions (NO_3_) in the plants, an aqueous solution of commercial potassium nitrate (KNO_3_) salts was used in equal molar amounts. This nitrate control for 1,600 mg/kg corresponds to 603.38 μmol of nitrate. Salt-free water was used as a negative control (0 mg Ni per 1 kg of dry soil). The experimental design was a completely randomized block design with 12 replications per each treatment.

Damages to plants were assessed every two days based on a rating scale of 1 to 9, 1 representing no visible toxicity symptoms and 9 was dead plants as described in [7]. Plants were rated individually and a genotype with a score of 1 to 3 was considered nickel resistant, 4 to 6, moderately resistant/susceptible, and 7 to 9 susceptible. Root samples were collected from the treated seedlings, frozen in liquid nitrogen, and stored at -20°C until future analyses. Total RNA was extracted from these samples and used for downstream transcriptome analyses.

### 2.2. RNA extraction

Total RNA was extracted using the Plant/Fungi Total RNA Purification kit from Norgen Biotek Corporation (Thorold, Canada). Samples were quantified with the Qubit® RNA BR Assay kit by Life Technologies (Carlsbad, United States). The RNA was run on a 1% agarose gel to verify its quality by confirming there were two sharp rRNA bands (28S and 18S) with no smearing/degradation.

### 2.3. RNA-seq and transcript alignment with a draft genome

RNA- seq libraries were created using the TruSeq RNA-Seq Sample Prep Kit and following manufacturer protocols (Illumina Inc., San Diego, CA). Messenger RNA (mRNA) was extracted from total RNA by selecting for RNA with poly-A tails. This fraction was chemically fragmented and then cDNA was synthesized in two steps (first strand and second strand). The cDNA ends were repaired, followed by the addition of adenosines to the 3’ ends and then adapter ligation. Complementary DNA (cDNA) fragments (with sizes of 200 ± 25 bp) were gel-purified and enriched by PCR. The Bioanalyzer 2100 (Agilent Technologies, Santa Clara, CA) was used to quantify libraries and then sequencing was done on the Illumina HiSeq 2000 sequencing system (Illumina Inc.) at Seq Matic (Fremont California, USA).

STAR (Spliced Transcripts Alignment to a Reference) software was used for alignment and annotation of raw RNA-seq data. Reads were aligned to the draft *Populus tremuloides* genome published by Lin *et al*. [12] using the two-pass method for increased sensitivity in detecting novel transcripts. All reads from the sequenced samples were used for alignment and merged into one for input into the StringTie software to detect transcripts based on existing gene annotation, novel transcripts, and new isoforms. The CDS (coding sequences) of the transcripts were further delineated with the transdecoder program. The resulting peptide sequences of transcripts were mapped to protein sequences in the Uniprot database (http://www.uniprot.org/) using RAPsearch2. Annotation and gene ontology information was assigned to transcripts based on the best match.

### 2.4. Gene expression and sampling QC

Transcript alignment bam files were mapped to the updated annotation file of the draft genome and the FeatureCounts tool from Subread was used to determine raw gene count. Additional QC was performed to determine the number of genes expressed. Genes with at least 1, 2, 10, 50 or 100 counts were considered. Generally, the number of genes with two or more counts provides a rough estimate of genes expressed. Genes with only one read could be noise. The number of genes with 10 or more reads was an indication of how many genes had sufficient reads to be included in downstream statistical analysis. Another QC measure applied to the data was to verify that the top 100 genes with highest number of reads did not make up a disproportionately high percentage (>35%) of all RNA-seq reads. This could indicate a bottlenecking issue that occurred during library preparation where only a few genes were amplified many times.

For downstream QC analysis, genes that had a counts per million (CPM) value ≥ 1 for at least two samples were considered. The gene count data was processed with the TMM normalization method from the edgeR package. Samples were scaled with a normalization factor to remove differences between the RNA population composition of each sample. An extreme normalization factor (>1.5 or <0.66) for a sample could indicate an outlier or a large biological difference compared to other samples.

Boxplots summarizing gene expression distribution were created by log-2 transforming the normalized gene counts with the voom method from the R Limma package. This method indicates outliers or samples with large biological differences if the distribution is high or low compared to other samples.

Sample relationships were assessed with a multidimensional plot created using the R Limma package. Ideally, the biological replicates would cluster together, and different treatment samples separate from each other. Hierarchical clustering of samples was further assessed with a heatmap created with the made4 (multivariate analysis of microarrays data using ADE4) package from R Limma. It included the top 5000 genes with variable expression. Genes were chosen with a standard deviation (SD) >30% of the mean expression values. If there were more than 5000 variable genes, then genes with mean logCPM <1 were removed. Remaining genes were ranked by SD/Mean and the top 5000 were chosen. Gene expression levels were plotted for each sample to identify overall patterns of sample clustering. Scatterplots between pairs of samples indicated how similar they were to each other. Biological replicates or samples from the same treatment should have high correlation values. One total nickel resistant sample (TotNi.R3) was identified as a potential outlier because gene expression was more like susceptible samples. It was removed from this treatment group for downstream gene expression analysis. Principle component analysis (PCA) and an overview gene expression heatmap confirmed this conjecture.

### 2.5. Differentially Expressed Genes (DEG) analysis

DEG analysis was performed between sample treatments. All the genes in the expressed genes QC list that have ≥ 10 raw counts in at least three samples were tested. The gene expression values were calculated as Reads Per Kilobase per Million reads mapped (RPKM). First raw read counts were converted for each gene to counts per million (cpm) to normalize for sequencing depth. The cpm was divided by gene length in kb to normalize for transcript length. For paired-end data, two paired reads were treated as one fragment, and the FPKM value was determined using the same calculation method. Differentially expressed genes were filtered through two stringency levels. The high stringency condition is the recommended standard cut-off of a two-fold change with an FDR (False Discovery Rate) value ≤ 0.05 and logFC value ≥1 (upregulated) or ≤ -1 (downregulated). The low stringency cutoff of a two-fold change and p-value ≤ 0.01 was applied to treatment comparisons with ≤ 10 genes that met the standard cutoff. Gene set enrichment analysis (GSEA) analysis was performed for the upregulated and downregulated genes to identify top GO categories enriched in the data. Only gene sets with a minimum of 10 genes and a maximum of 1000 genes were included. The annotated gene set of all the DEGs identified for the nickel resistant vs. susceptible comparison was run through the Plant-Slim function and then GO category graphs were created with the OmicsBox BLAST2GO program. The percentage of differentially expressed transcripts distributed within each of the three GO function categories (Biological Process, Molecular Function and Cellular Component) was calculated. All of the annotated genes from the water treatment were also run through Plant-Slim and GO category graphs were created to provide a summary of GO functions for the *Populus tremuloides* control.

## 3. Results

### 3.1. Assessment of nickel toxicity on *P*. *tremuloides* seedlings

**Fig 1:**
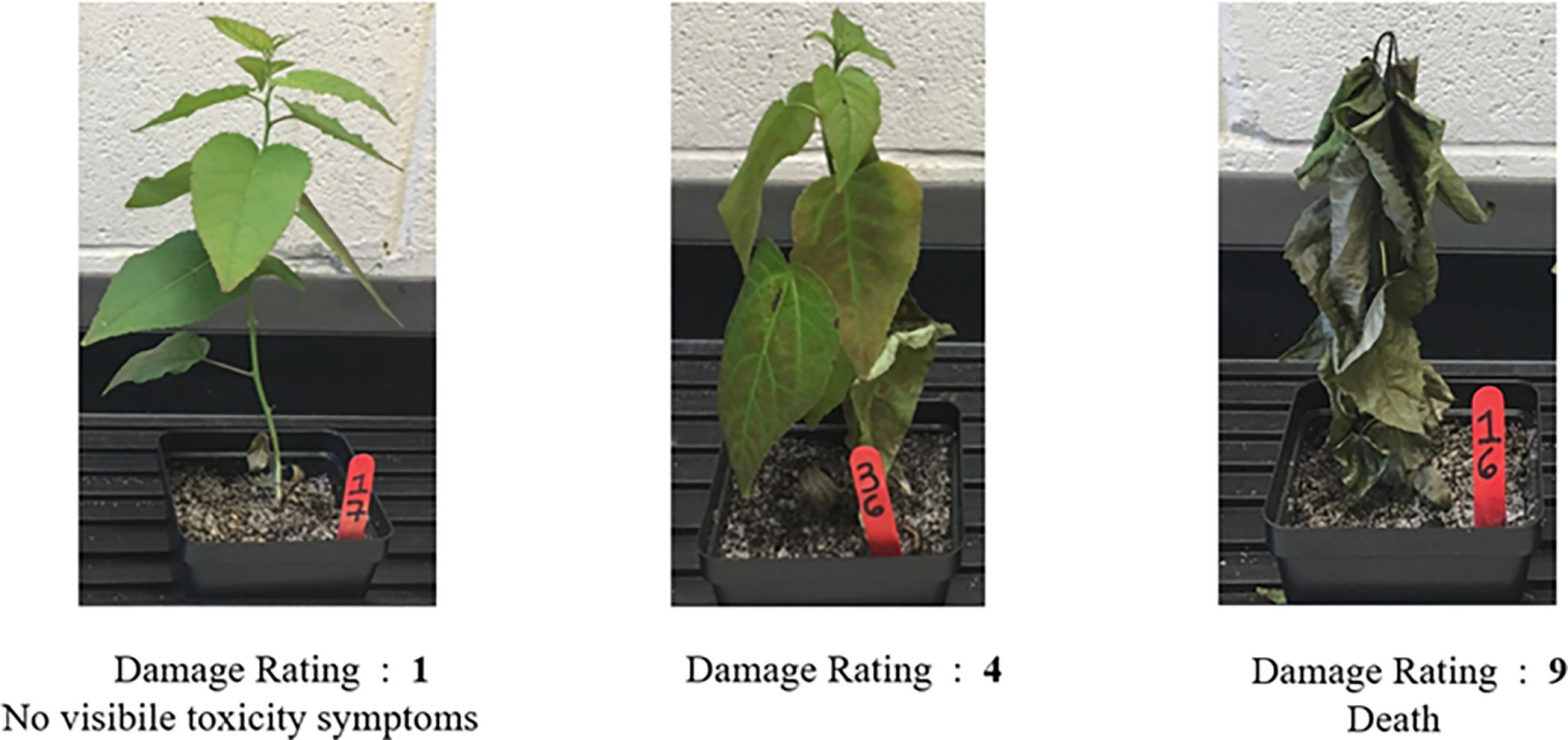
A nickel-resistant (left), moderately nickel-resistant (middle) and nickel-susceptible (right) *P*. *tremuloides* seedling on day seven (7) after treatment with 1,600 mg/kg nickel nitrate.

Significant differences were observed among genotypes treated with 1,600 mg/kg Ni dose. Some plants were dead or nearly dead by the end of the experiment (day 7). This treatment showed the highest variation in plant reactions to Ni salts where after day 7, there were three resistant, two moderately susceptible and seven susceptible plants (Fig 1). Plants treated with potassium nitrate salts used as control showed no significant damages.

### 3.2. Transcript assembly and QC analysis

Sequencing generated 27–45 million 2X150 paired-end reads of raw data per sample. The alignment performed with StringTie Software added two groups of transcripts to the draft genome annotation. Group 1 contained 32,677 new isoforms that match to 17,254 genes. Group 2 contained 17,349 novel transcripts that represent 16,157 novel genes. The second group of novel transcripts representing 16,157 novel genes added to the draft annotation may contain mostly non-coding RNAs as indicated by the limited amount of long gene coding regions.

A total of 52,987 genes were identified and considered for downstream QC analysis. After normalization, samples generally met the QC test standards. The Ni susceptible plants have an overall smaller number of genes expressed. No potential outlier from the 18 samples was detected in the gene expression and sampling QC analysis. An MDS plot (multi-dimensional plot) made for sample clustering analysis did suggest that one nickel resistant sample (TotNi.3) appeared more like a nickel susceptible sample. Additional measures like the variable gene expression heat map were consistent with this observation. This sample was considered a treatment group outlier and was removed from the nickel resistant group for the resistant-susceptible genotype comparison.

The entire *P*. *tremuloides* transcriptome dataset was deposited in the DDBJ/EMBL/GenBank under the BioProject accession number PRJNA820879.

### 3.3. Gene ontology classification of *Populus tremuloides*

**Fig 2:**
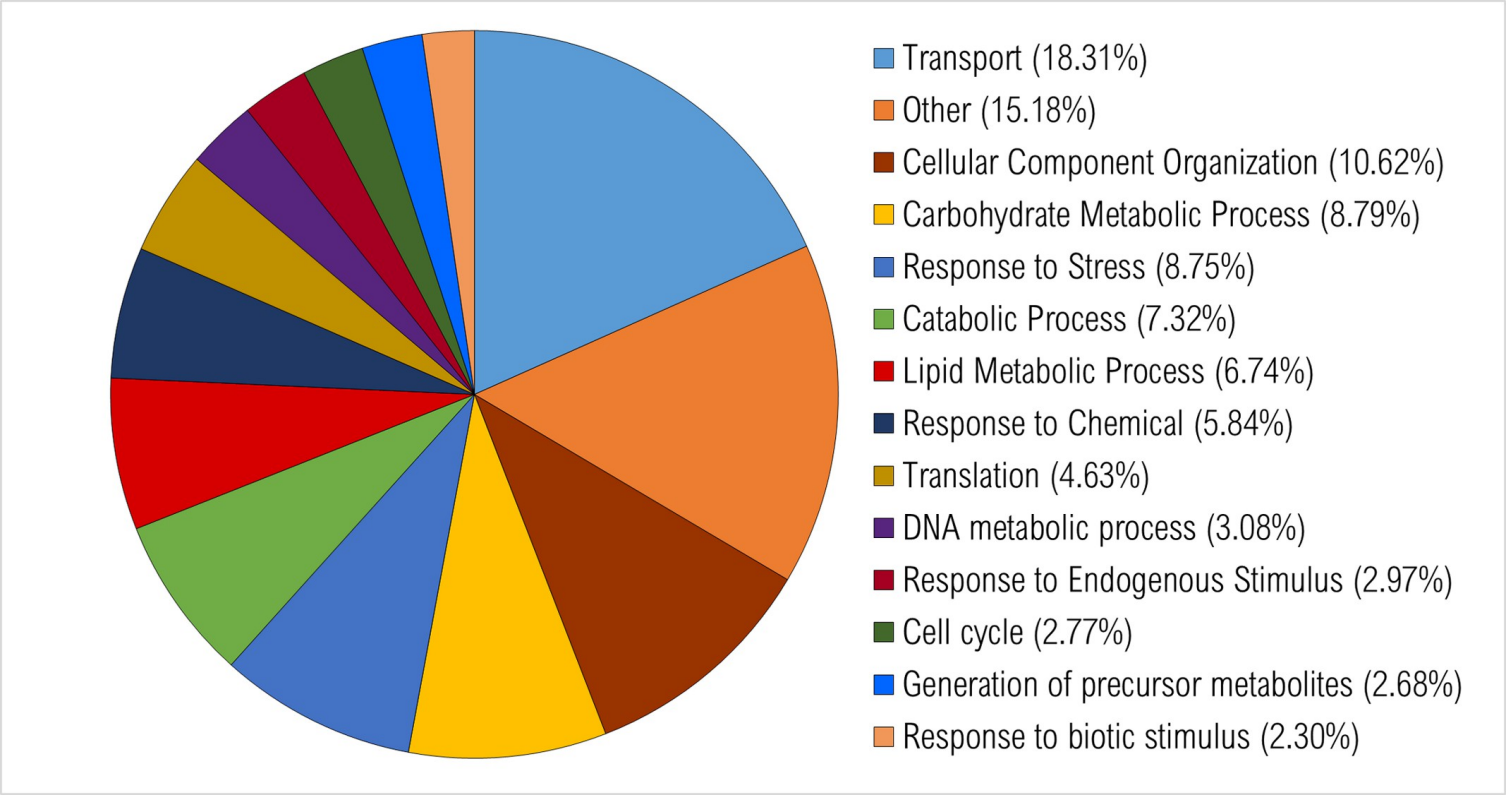
Percentage of transcripts in *Populus tremuloides* control samples. A total of 9766 transcripts were assigned ontology and grouped by biological processes using BLAST2GO. Categories under 2% were grouped together and classified as “other”.

**Fig 3:**
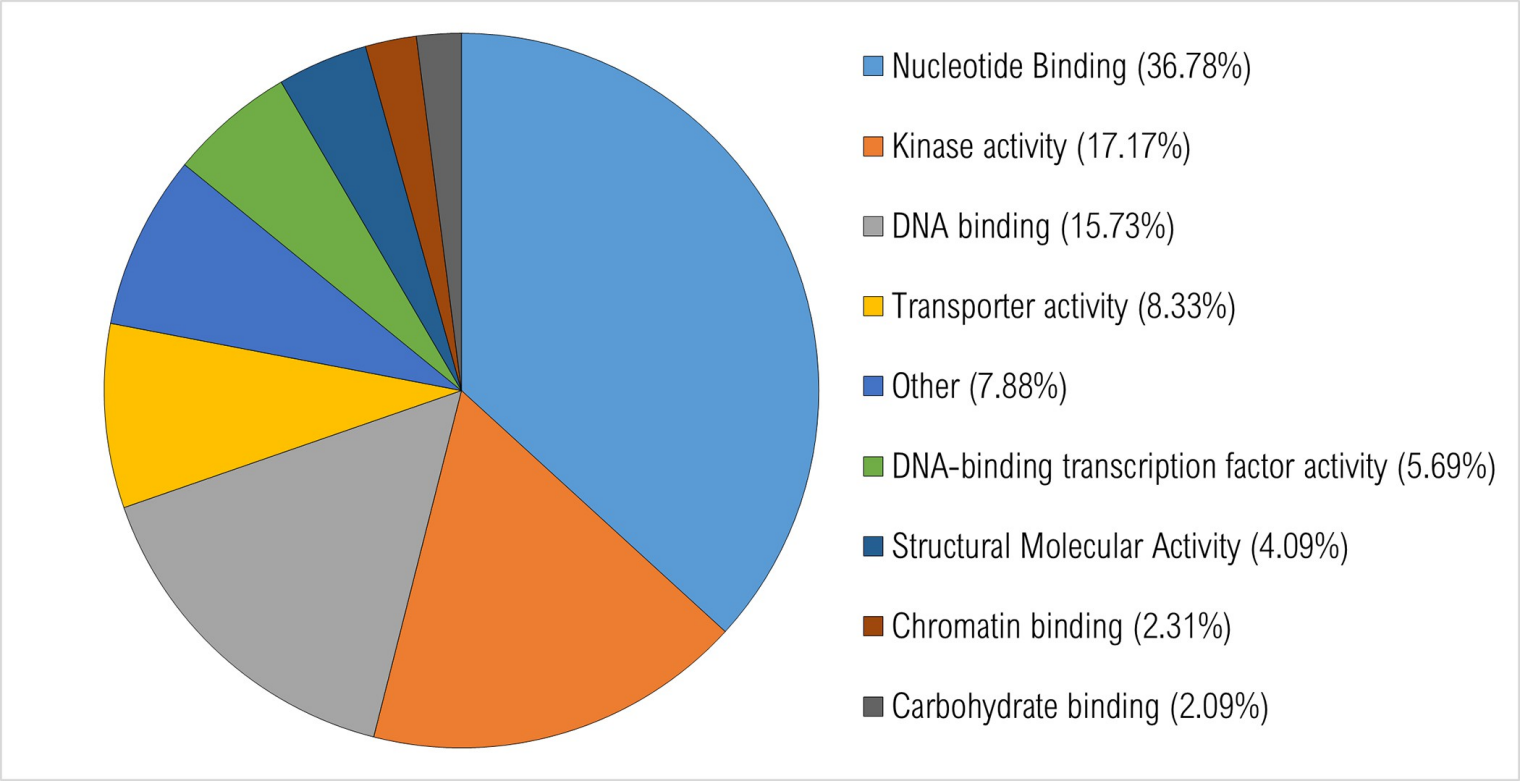
Percentage of transcripts in *Populus tremuloides* control samples. A total of 9953 transcripts were assigned ontology and grouped by molecular function using BLAST2GO. Categories under 2% were grouped together and classified as “other”.

**Fig 4:**
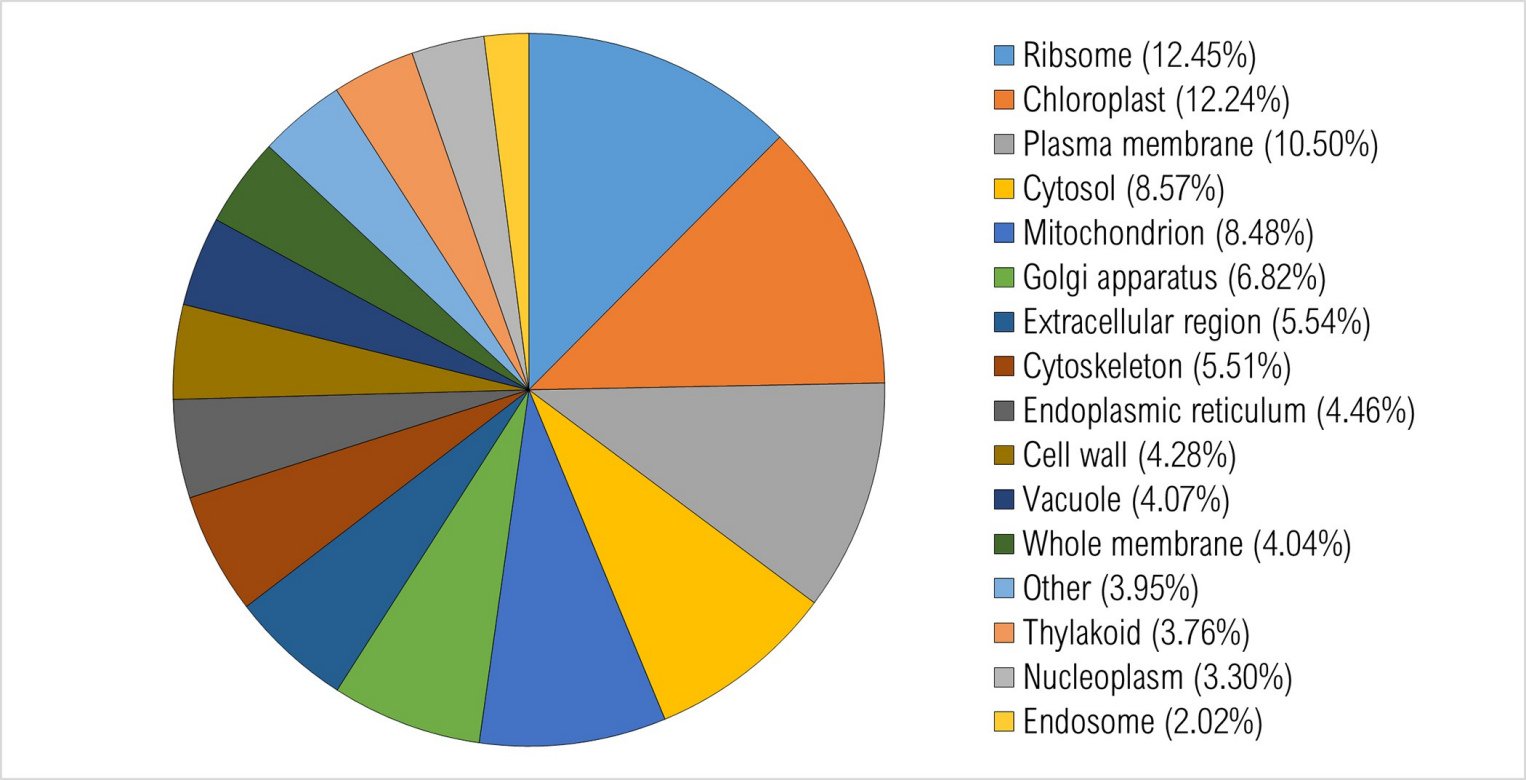
Percentage of transcripts in *Populus tremuloides* control samples. A total of 3268 transcripts were assigned ontology and grouped by cellular compartment using BLAST2GO. Categories under 2% were grouped together and classified as “other”.

All expressed annotated transcripts were run through BLAST2GO. Gene ontology was assigned, and graphs were generated to display the distribution of expressed transcripts for biological function, molecular function, and cell compartmentalization. In general, the number of transcripts identified for each different group (control, resistant, susceptible) were similar. GO category graphs for all annotated genes expressed in the water control treatment plants are displayed in Figs 2–4.

A total of 9,766 transcripts were assigned ontology for biological function. About 66.3% of all transcripts were involved in the transport, cellular component organization (CCO), carbohydrate metabolic process (CMP), response to stress, catabolic process, lipid metabolic process and response to chemical categories (Fig 2).

For molecular function, 9,953 transcripts were assigned ontology. About 37% of transcripts code for proteins involved in nucleotide binding activities, 17.1% for kinase activities, 15.7% for DNA binding activities and 8.3% for transporter activities (Fig 3).

For cellular compartmentalization, 3,268 transcripts were assigned ontology. Overall, 12.5% of transcripts were localized to the ribosome, 12.2% in chloroplast, 10.5% in plasma membrane, 8.6% in cytosol, 8.5% in mitochondria, 6.8% in Golgi apparatus, 5.5% in extracellular region, and 5.5% in cytoskeleton (Fig 4).

Altogether, most of the expressed transcripts among these three principal gene ontologies were distributed into the categories of CCO, CMP, nucleotide binding, kinase and DNA binding activities, ribosome, chloroplast, cytosol, and mitochondria. These functional processes are likely majorly involved in typical *P*. *tremuloides* gene activities.

### 3.4. Differentially expressed gene analysis

A total of 36,770 genes from the list of 52,987 genes were chosen as expressed for DEG analysis. These met the cut-off of ≥ 10 raw gene counts in at least three samples. The average expression values of these genes were compared in nickel resistant and nickel susceptible genotypes. With the high stringency (two-fold change, FDR value ≤ 0.05 and logFC value ≥1 (upregulated) or ≤ -1 (downregulated), 2,128 genes were upregulated, and 762 genes were downregulated in nickel resistant genotypes. With the low stringency filter (two-fold change and p-value ≤ 0.01), 3,070 genes were upregulated, and 1,186 genes were downregulated. GSEA analysis for these up- and down-regulated genes from the high stringency conditions was performed and 721 gene sets out of 3422 were used for analysis after filtering for gene set size. From these gene sets, 575 were upregulated and 146 were downregulated in nickel resistant phenotypes compared to susceptible genotypes.

Overall, the top upregulated GO functions in resistant samples were involved with the kinesin complex, microtubule-based movement, and cell wall modification (S1 Fig). The top down-regulated functions were DNA integration, regulation of growth and negative regulation of programmed cell death (S2 Fig).

### 3.5. Pairwise comparison of resistant and susceptible genotypes

**Fig 5:**
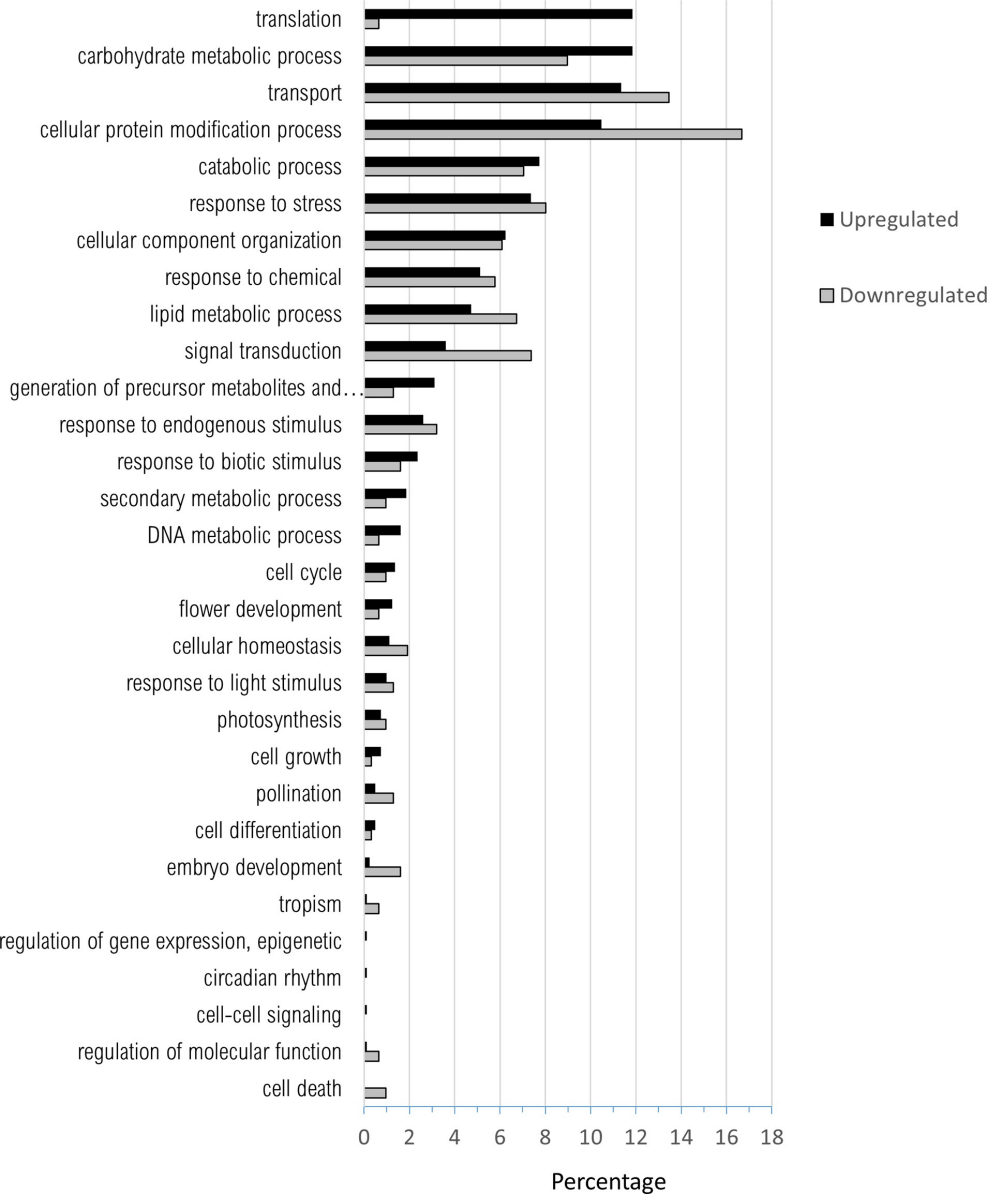
Percentage of upregulated (total number = 802) and downregulated (total number = 312) when nickel resistant *Populus tremuloides* genotypes were compared to nickel susceptible genotypes. Transcripts were assigned gene ontology and grouped by Biological Process using BLAST2GO (Omics Box).

**Fig 6:**
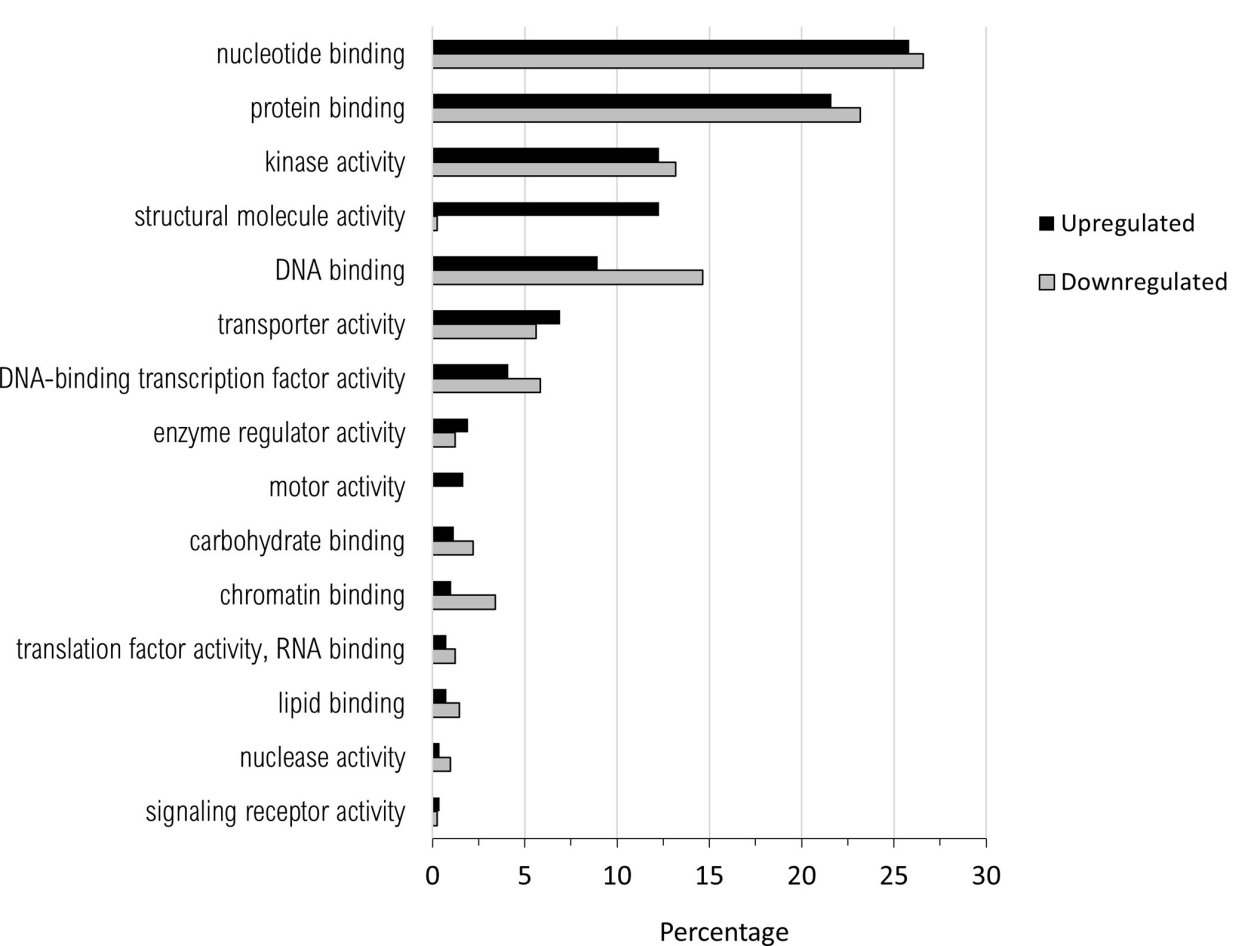
Percentage of upregulated (total number = 782) and downregulated (total number = 410) when nickel resistant *Populus tremuloides* genotypes were compared to nickel susceptible genotypes. Transcripts were assigned gene ontology and grouped by Molecular Function using BLAST2GO (Omics Box).

**Fig 7:**
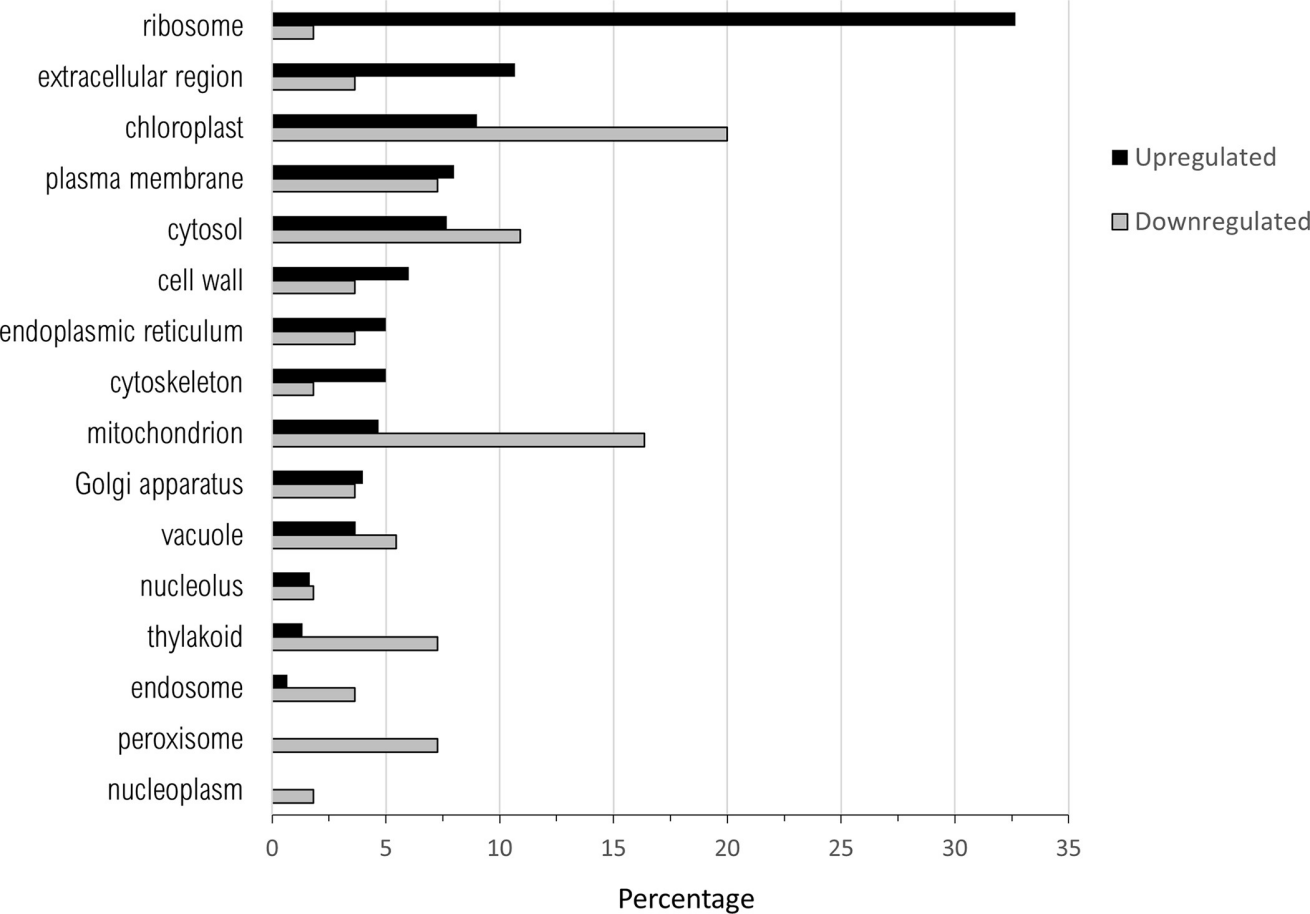
Percentage of upregulated (total number = 300) and downregulated (total number = 55) when nickel resistant *Populus tremuloides* genotypes were compared to nickel susceptible genotypes. Transcripts were assigned gene ontology and grouped by Cellular Component using BLAST2GO (Omics Box).

There were no significant differentially expressed genes (DEGs) when nickel-resistant genotypes were compared to the water control using the high stringency cut-off. Using these same statistical requirements, the nickel-susceptible treatment group did have significant differences in gene expression when compared to nickel-resistant or the water control. The list of differentially regulated genes found in the pairwise comparison of nickel-resistant and susceptible transcriptomes was filtered through the three principle GO ontology categories (Figs 5–7).

There was a higher number of total upregulated transcripts over downregulated for all three GO functions. Upregulation indicates increased expression of genes in RGs and downregulation indicates genes with increased expression in SGs.

For biological process (Fig 5), the highest percentages of upregulated transcripts were in the categories of translation (11.8%), carbohydrate metabolic process (11.8%), transport (11.3%), cellular protein modification process (10.4%), and catabolic process (8.0%). The highest percentages of downregulated transcripts were in the categories of cellular protein modification process (16.7%), transport (13.4%), carbohydrate metabolic process (9.0%), response to stress (8.0%) and signal transduction (7.3%). When comparing between up and downregulation, translation had a much higher percentage of transcripts that were upregulated than downregulated. Carbohydrate metabolic process also had more upregulated genes in RS genotypes. Transport, cellular protein modification process, lipid metabolic process and signal transduction all had a noticeably increased percentage of downregulated transcripts over upregulated.

For molecular function (Fig 6), the highest percentage of upregulated transcripts were in the categories of nucleotide binding (25.8%), protein binding (21.6%), kinase activity (12.3%), structural molecule activity (12.3%) and DNA binding (9.0%). Most downregulated transcripts coded for proteins involved in nucleotide binding (26.6%), protein binding (23.2%), DNA binding (14.6%) and kinase activity(13.2%). Almost all differentially expressed transcripts for structural molecule activity were upregulated (12.3%), with only 0.24% of downregulated transcripts. DNA binding transcripts were more downregulated (14.6%) than upregulated (9.0%).

For cellular compartment localization (Fig 7), almost a third of upregulated transcripts were in the ribosome (32.7%). The categories with the next highest percentage of upregulated transcripts are extracellular region (10.7%), chloroplast (9%), plasma membrane (8.0%) and cytosol (7.7%). The cellular compartment associated with downregulated transcripts was more variably distributed across categories. The highest percentages of downregulated transcripts in RG localized to the chloroplast (20.0%), mitochondrion (16.4%), and cytosol (10.9%). Most or all differentially expressed transcripts identified in the thylakoid, peroxisome, endosome, and nucleoplasm were downregulated in RGs.

### 3.6. Characterization of highly differentially expressed genes

**Fig 8:**
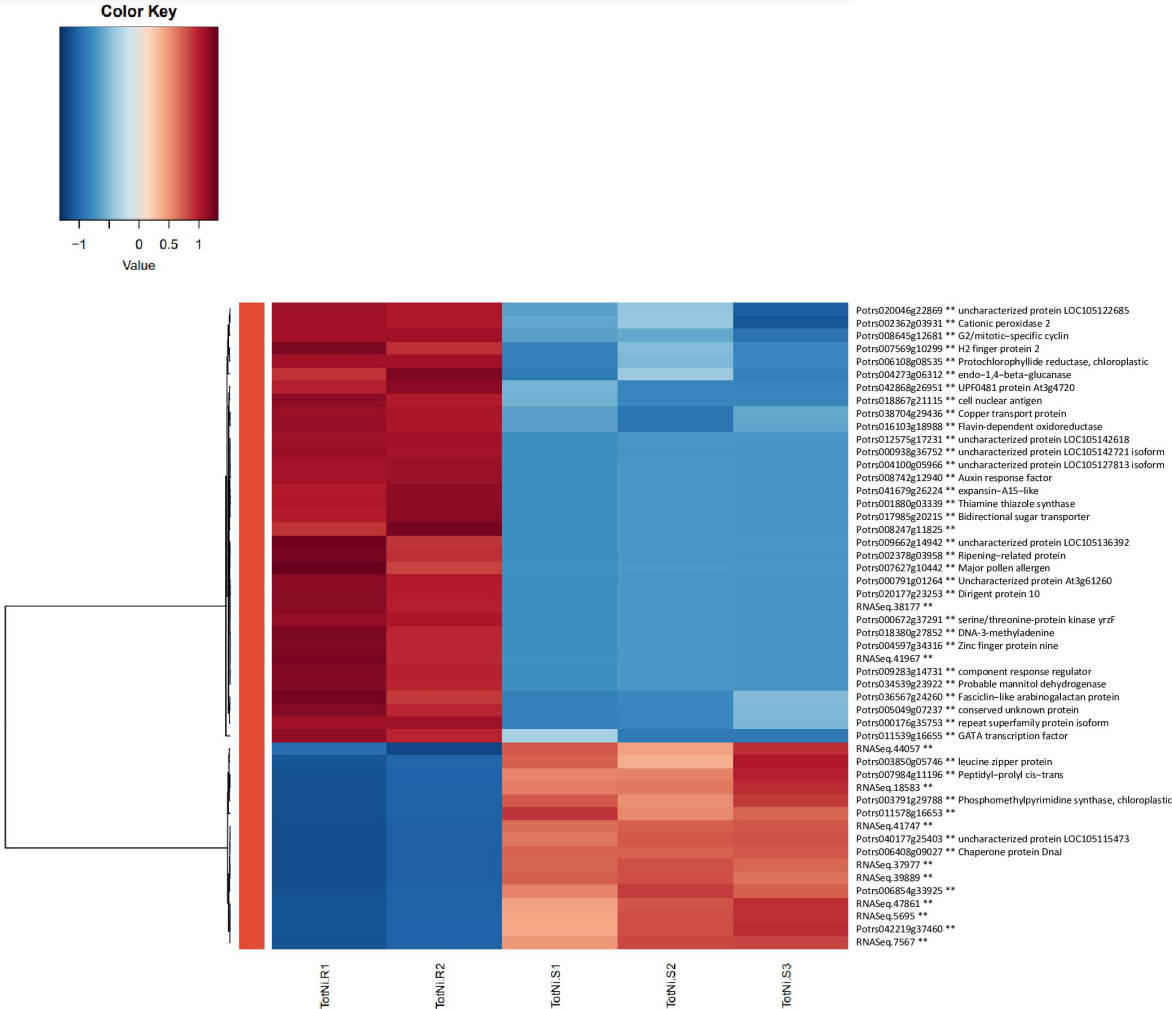
Top 50 differentially expressed genes between nickel resistant and susceptible *Populus tremuloides* based on LogFC. The red colour represents an upregulation and downregulation is blue.

**Fig 9:**
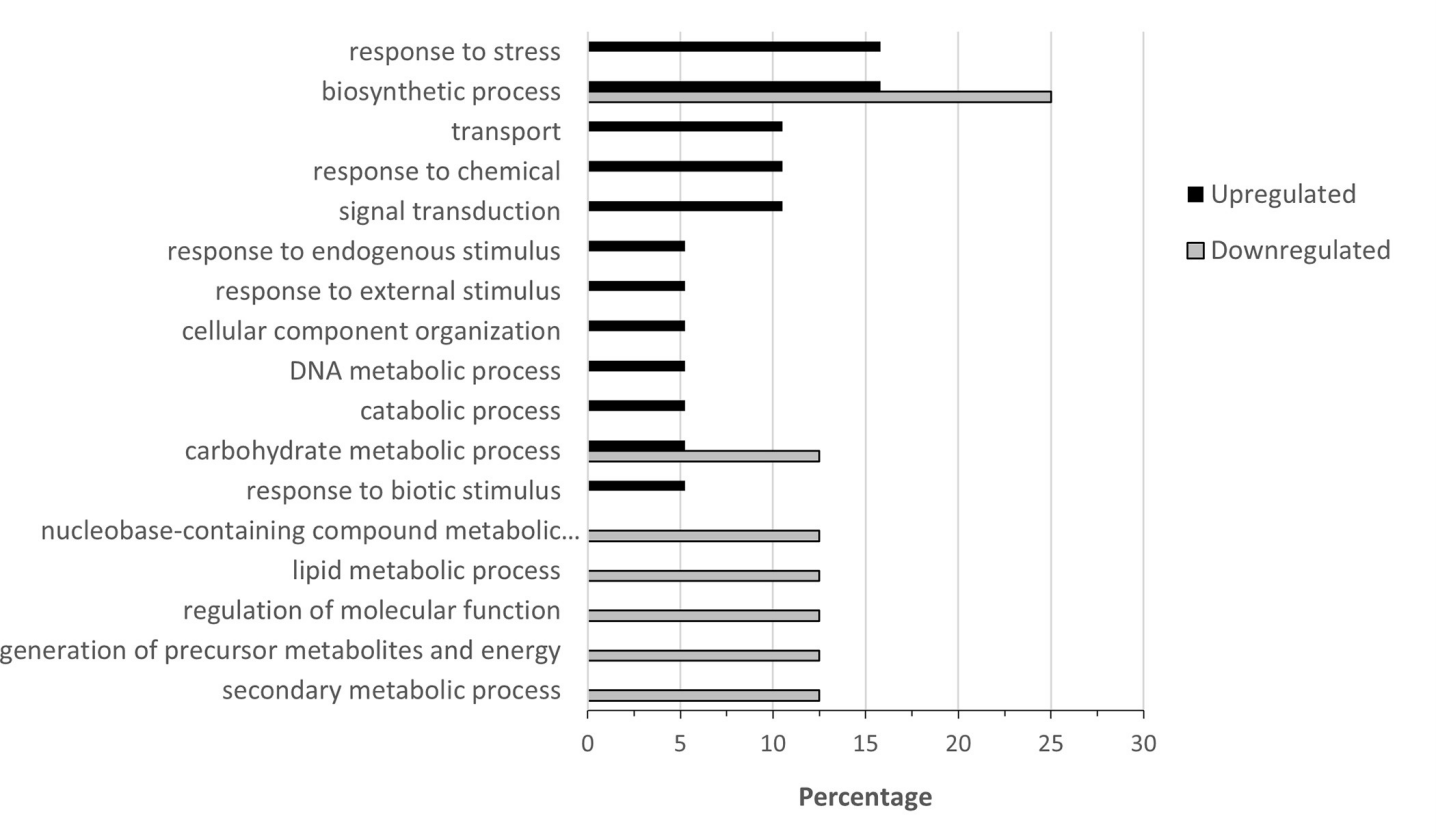
Percentage of upregulated (n = 19) and downregulated (n = 8) transcripts from the Top 50 differentially expressed genes (DEGs) when *Populus Tremuloides* with nickel resistant genotypes were compared to nickel susceptible genotypes. Only transcripts with assigned gene ontology were included and these were grouped by biological process using BLAST2GO (Omics Box).

**Fig 10:**
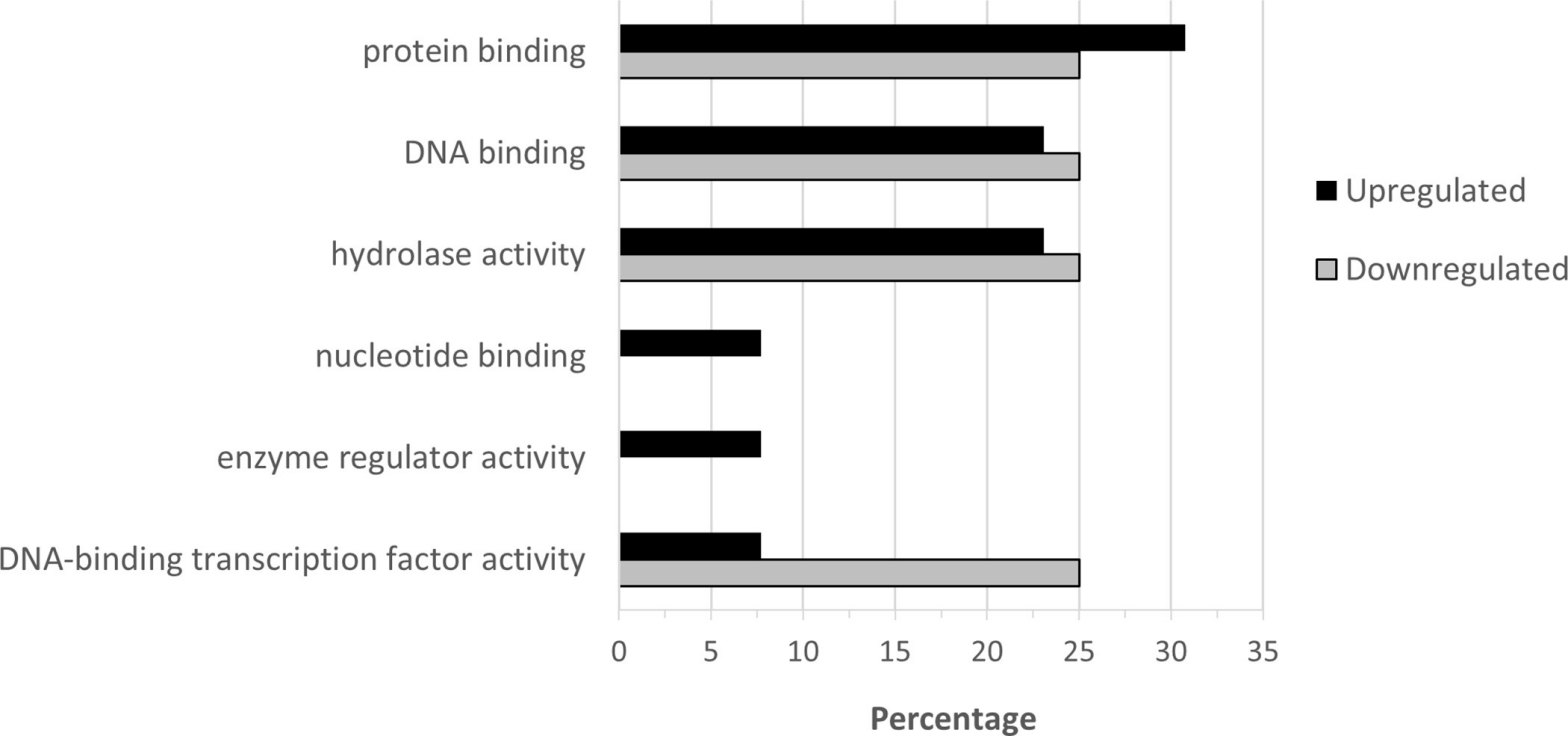
Percentage of upregulated (n = 13) and downregulated (n = 4) transcripts from the Top 50 differentially expressed genes (DEGs) when *Populus Tremuloides* nickel resistant genotypes were compared to nickel susceptible genotypes. Only transcripts with assigned gene ontology were included and these were grouped by molecular function using BLAST2GO (Omics Box).

**Fig 11:**
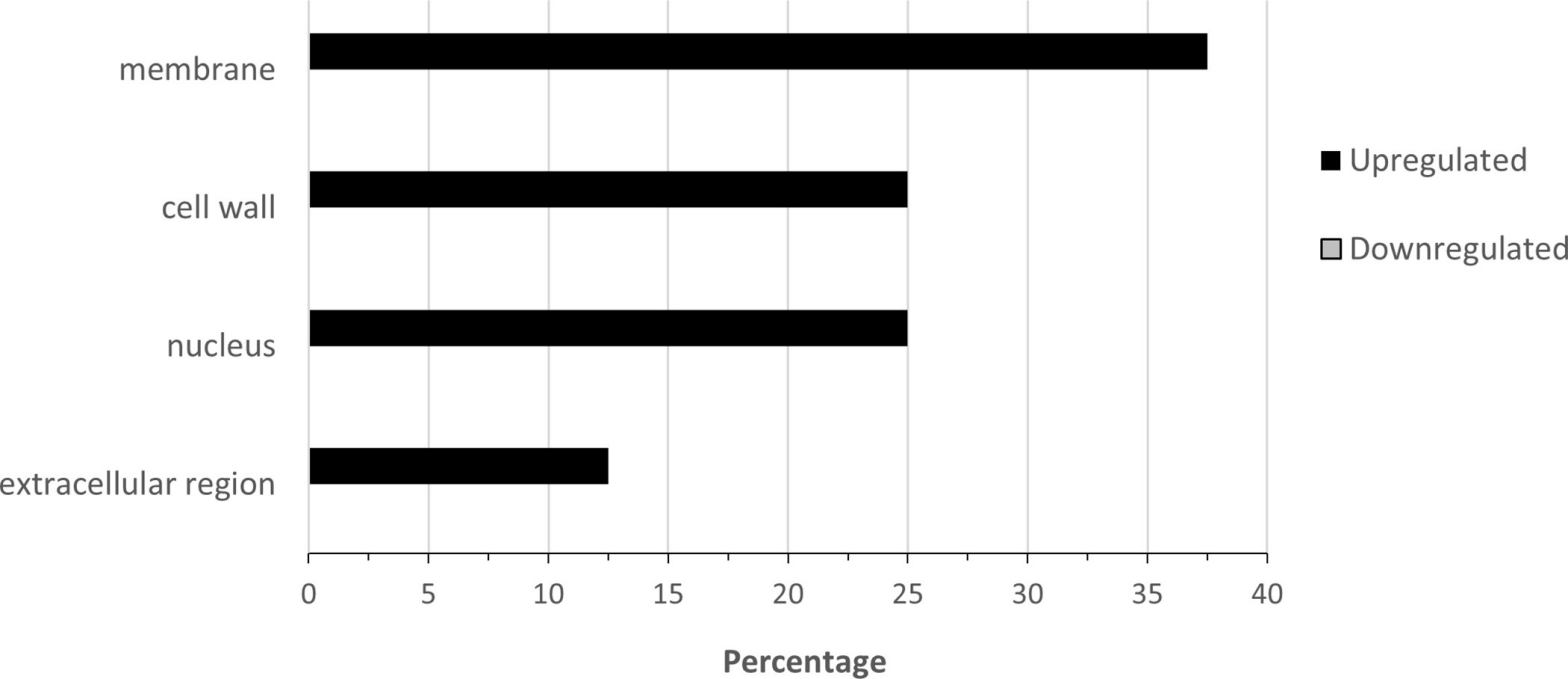
Percentage of upregulated (n = 8) and downregulated (n = 0) transcripts from the Top 50 differentially expressed genes (DEGs) when nickel resistant *Populus Tremuloides* genotypes were compared to nickel susceptible genotypes. Only transcripts with assigned gene ontology were included and these were grouped by cellular component using BLAST2GO (Omics Box).

The heatmap in Fig 8 displays the patterns of gene expression for the top 50 differentially expressed genes (DEGs) when resistant genotypes (RGs) were compared to susceptible genotypes (SGs). Genes were ranked based on LogFC expression values and the list of the top 50 DEGs is shown in Table 1. The majority of top DEGs were upregulated in RGs (n = 34). Most of the downregulated genes (n = 16) lack assigned gene ontology and description. This list of 50 genes was filtered through the three principal gene ontology categories to analyze the distribution of transcript functions (Figs 9–11).

**Table 1:** Top 50 differentially expressed genes (up or downregulated) in resistant trembling aspen (*Populus tremuloides*) genotypes compared to susceptible trembling aspen genotypes based on LogFC.

Plants (RPKM)									
Rank	geneID	Res. 1	Res. 2	Sus. 1	Sus. 2	Sus. 3	logFC	adj.P.Val	Description
1	RNASeq.41967	179.84	62.86	0.00	0.00	0.00	10.25	0.0205	
2	Potrs000938g36752	54.47	45.35	0.00	0.00	0.00	10.05	0.0205	uncharacterized protein LOC105142721 isoform
3	Potrs018867g21115	40.42	24.44	0.05	0.00	0.00	9.72	0.0205	cell nuclear antigen
4	Potrs020177g23253	21.77	12.05	0.00	0.00	0.00	9.65	0.0205	Dirigent protein 10
5	Potrs011539g16655	31.36	16.73	0.11	0.00	0.00	9.62	0.0208	GATA transcription factor
6	Potrs006108g08535	15.46	15.95	0.00	0.03	0.00	9.54	0.0205	Protochlorophyllide reductase, chloroplastic
7	Potrs004597g34316	13.50	5.75	0.00	0.00	0.00	9.51	0.0205	Zinc finger protein
8	Potrs000176g35753	9.08	9.96	0.00	0.00	0.02	9.49	0.0205	repeat superfamily protein isoform
9	Potrs002362g03931	50.73	41.35	0.08	0.19	0.00	9.48	0.0206	Cationic peroxidase 2
10	Potrs034539g23922	13.90	6.37	0.00	0.00	0.00	9.47	0.0205	Probable mannitol dehydrogenase
11	Potrs005049g07237	18.97	8.64	0.00	0.00	0.03	9.44	0.0205	conserved unknown protein
12	Potrs007627g10442	31.58	5.97	0.00	0.00	0.00	9.30	0.0216	Major pollen allergen
13	Potrs016103g18988	19.71	15.10	0.03	0.00	0.03	9.28	0.0205	Flavin-dependent oxidoreductase
14	RNASeq.38177	62.98	33.40	0.00	0.00	0.00	9.27	0.0205	
15	Potrs008742g12940	3.22	3.88	0.00	0.00	0.00	9.26	0.0205	Auxin response factor
16	Potrs004100g05966	12.75	15.25	0.00	0.00	0.00	9.25	0.0205	uncharacterized protein LOC105127813 isoform
17	Potrs000791g01264	11.80	7.13	0.00	0.00	0.00	9.25	0.0205	Uncharacterized protein At3g61260
18	Potrs017985g20215	8.34	15.81	0.00	0.00	0.00	9.19	0.0205	Bidirectional sugar transporter
19	Potrs001880g03339	6.54	11.01	0.00	0.00	0.00	9.19	0.0205	Thiamine thiazole synthase
20	Potrs008247g11825	12.45	43.60	0.00	0.00	0.00	9.18	0.0206	
21	Potrs041679g26224	7.36	12.51	0.00	0.00	0.00	9.18	0.0205	expansin-A15-like
22	Potrs036567g24260	28.65	7.94	0.00	0.00	0.04	9.18	0.0224	Fasciclin-like arabinogalactan protein
23	Potrs009283g14731	6.60	3.29	0.00	0.00	0.00	9.18	0.0205	component response regulator
24	Potrs038704g29436	10.28	8.62	0.01	0.00	0.02	9.15	0.0205	Copper transport protein
25	Potrs020046g22869	48.15	40.56	0.08	0.19	0.00	9.15	0.0205	uncharacterized protein LOC105122685
26	Potrs002378g03958	33.57	11.70	0.00	0.00	0.00	9.12	0.0205	Ripening-related protein
27	Potrs000672g37291	13.88	10.00	0.00	0.00	0.00	9.09	0.0205	serine/threonine-protein kinase yrzF
28	Potrs012575g17231	7.95	7.88	0.00	0.00	0.00	9.08	0.0205	uncharacterized protein LOC105142618
29	Potrs009662g14942	12.67	3.63	0.00	0.00	0.00	9.08	0.0205	uncharacterized protein LOC105136392
30	Potrs004273g06312	5.97	18.58	0.00	0.05	0.00	9.06	0.0244	endo-1,4-beta-glucanase
31	Potrs042868g26951	6.37	12.07	0.02	0.00	0.00	9.04	0.0205	UPF0481 protein At3g47200
32	Potrs007569g10299	54.17	20.11	0.00	0.10	0.00	9.03	0.0206	H2 finger protein
33	Potrs018380g27852	5.81	2.48	0.00	0.00	0.00	9.03	0.0205	DNA-3-methyladenine
34	Potrs008645g12681	10.10	10.89	0.02	0.02	0.00	9.00	0.0205	G2/mitotic-specific cyclin
35	RNASeq.7567	0.00	0.00	15.24	51.49	51.02	-8.99	0.0216	
36	RNASeq.41747	0.00	0.00	37.07	47.26	50.81	-9.01	0.0205	
37	Potrs006408g09027	0.00	0.00	25.59	25.82	28.22	-9.02	0.0205	Chaperone protein DnaJ
38	Potrs040177g25403	0.00	0.00	9.97	15.87	17.04	-9.03	0.0205	uncharacterized protein LOC105115473
39	Potrs007984g11196	0.00	0.00	7.02	7.41	29.68	-9.17	0.0231	Peptidyl-prolyl cis-trans
40	Potrs003791g29788	0.00	0.00	3.57	1.59	5.28	-9.30	0.0206	Phosphomethylpyrimidine synthase, chloroplastic
41	RNASeq.44057	0.12	0.00	86.30	28.90	157.76	-9.32	0.0407	
42	Potrs003850g05746	0.00	0.00	10.75	3.01	31.48	-9.33	0.0347	leucine zipper protein
43	Potrs006854g33925	0.00	0.00	29.96	86.88	48.55	-9.36	0.0205	
44	RNASeq.18583	0.00	0.00	37.90	41.03	125.39	-9.42	0.0215	
45	RNASeq.47861	0.00	0.00	24.27	86.94	146.15	-9.63	0.0321	
46	RNASeq.5695	0.00	0.00	5.71	22.67	34.63	-9.64	0.0335	
47	RNASeq.37977	0.00	0.00	42.63	57.91	39.58	-9.67	0.0205	
48	Potrs042219g37460	0.00	0.00	32.95	151.79	246.68	-9.74	0.0423	
49	RNASeq.39889	0.00	0.00	23.90	30.91	16.71	-10.12	0.0205	
50	Potrs011578g16653	0.00	0.00	55.19	14.19	25.08	-10.14	0.0231	

For Biological process, most categories consisted of only upregulated or downregulated genes (Fig 9). The exception is in biosynthetic process with three upregulated and two downregulated genes, and carbohydrate metabolic process with one upregulated and one downregulated gene. Other upregulated genes were associated with response to stress (3 genes), transport, response to chemical, signal transduction (2 genes each), response to endogenous stimulus, response to external stimulus, cellular component organization, DNA metabolic process and catabolic process (1 gene each). There was one downregulated gene each in secondary metabolic process, generation of precursor metabolites and energy, regulation of molecular function, lipid metabolic process and nucleobase-containing compound metabolic process.

For molecular function, the highest percentage of upregulated genes are involved in protein binding with four identified genes (Fig 10). Hydrolase activity and DNA binding each had three upregulated genes. Nucleotide binding, enzyme regulator activity and DNA-binding transcription factor activity each had one upregulated gene. There were only four downregulated genes with annotations, and they were distributed evenly within protein binding, DNA binding, hydrolase activity and DNA-binding transcription factor activity.

There was limited gene ontology info for cellular component (Fig 11). From the eight upregulated genes identified, three were localized to the nucleus, two to extracellular region, two to cell membrane and one to cell wall. There were no downregulated genes with cellular component data.

The Top 30 upregulated and downregulated genes identified in resistant genotypes are displayed in Tables 2 and 3 respectively. Specifically, some of the highly upregulated genes in RG were characterized as Dirigent protein 10, GATA transcription factor, RuBisCO reductase (chloroplastic), Zinc finger protein, Cationic peroxidase 2, Probable mannitol dehydrogenase, Flavin-dependent oxidoreductase, Auxin response factor, Bidirectional sugar transporter, thiamine thiazole synthase, and DNA3-methyladenine.

**Table 2:** Top 30 upregulated transcripts in resistant trembling aspen (*Populus tremuloides*) genotypes compared to susceptible trembling aspen genotypes based on LogFC.

		Plants (RPKM)							
Rank	Gene ID	Res. 1	Res. 2	Sus. 1	Sus. 2	Sus. 3	logFC	adj.P.Val	Description
1	RNASeq.41967	179.84	62.86	0.00	0.00	0.00	10.25	0.0205	
2	Potrs000938g36752	54.47	45.35	0.00	0.00	0.00	10.05	0.0205	uncharacterized protein LOC105142721 isoform
3	Potrs018867g21115	40.42	24.44	0.05	0.00	0.00	9.72	0.0205	cell nuclear antigen
4	RNASeq.9446	87.68	11.05	0.00	0.00	0.00	9.72	0.0300	
5	Potrs020177g23253	21.77	12.05	0.00	0.00	0.00	9.65	0.0205	Dirigent protein 10
6	Potrs011539g16655	31.36	16.73	0.11	0.00	0.00	9.62	0.0208	GATA transcription factor
7	Potrs006108g08535	15.46	15.95	0.00	0.03	0.00	9.54	0.0205	Protochlorophyllide reductase, chloroplastic
8	Potrs004597g34316	13.50	5.75	0.00	0.00	0.00	9.51	0.0205	Zinc finger protein
9	Potrs000176g35753	9.08	9.96	0.00	0.00	0.02	9.49	0.0205	repeat superfamily protein isoform
10	Potrs002362g03931	50.73	41.35	0.08	0.19	0.00	9.48	0.0206	Cationic peroxidase 2
11	Potrs034539g23922	13.90	6.37	0.00	0.00	0.00	9.47	0.0205	Probable mannitol dehydrogenase
12	Potrs005049g07237	18.97	8.64	0.00	0.00	0.03	9.44	0.0205	conserved unknown protein
13	Potrs007627g10442	31.58	5.97	0.00	0.00	0.00	9.30	0.0216	Major pollen allergen
14	Potrs016103g18988	19.71	15.10	0.03	0.00	0.03	9.28	0.0205	Flavin-dependent oxidoreductase
15	RNASeq.38177	62.98	33.40	0.00	0.00	0.00	9.27	0.0205	
16	Potrs008742g12940	3.22	3.88	0.00	0.00	0.00	9.26	0.0205	Auxin response factor
17	Potrs004100g05966	12.75	15.25	0.00	0.00	0.00	9.25	0.0205	uncharacterized protein LOC105127813 isoform
18	Potrs000791g01264	11.80	7.13	0.00	0.00	0.00	9.25	0.0205	Uncharacterized protein At3g61260
19	Potrs017985g20215	8.34	15.81	0.00	0.00	0.00	9.19	0.0205	Bidirectional sugar transporter
20	Potrs001880g03339	6.54	11.01	0.00	0.00	0.00	9.19	0.0205	Thiamine thiazole synthase
21	Potrs008247g11825	12.45	43.60	0.00	0.00	0.00	9.18	0.0206	
22	Potrs041679g26224	7.36	12.51	0.00	0.00	0.00	9.18	0.0205	expansin-A15-like
23	Potrs036567g24260	28.65	7.94	0.00	0.00	0.04	9.18	0.0224	Fasciclin-like arabinogalactan protein
24	Potrs009283g14731	6.60	3.29	0.00	0.00	0.00	9.18	0.0205	component response regulator
25	Potrs038704g29436	10.28	8.62	0.01	0.00	0.02	9.15	0.0205	Copper transport protein
26	Potrs020046g22869	48.15	40.56	0.08	0.19	0.00	9.15	0.0205	uncharacterized protein LOC105122685
27	Potrs002378g03958	33.57	11.70	0.00	0.00	0.00	9.12	0.0205	Ripening-related protein
28	Potrs000672g37291	13.88	10.00	0.00	0.00	0.00	9.09	0.0205	serine/threonine-protein kinase yrzF
29	Potrs018426g20661	2.57	9.89	0.02	0.00	0.00	9.09	0.0320	ABC transporter G family member
30	Potrs012575g17231	7.95	7.88	0.00	0.00	0.00	9.08	0.0205	uncharacterized protein LOC105142618

**Table 3:** Top 30 downregulated transcripts in resistant trembling aspen (*Populus tremuloides*) genotypes compared to susceptible trembling aspen genotypes based on LogFC.

		Plants (RPKM)							
Rank	geneID	Res. 1	Res. 2	Sus. 1	Sus. 2	Sus. 3	logFC	adj.P.Val	Description
1	Potrs011578g16653	0.00	0.00	55.19	14.19	25.08	-10.14	0.0231	Probable Carboxylesterase 13
2	RNASeq.39889	0.00	0.00	23.90	30.91	16.71	-10.12	0.0205	
3	Potrs042219g37460	0.00	0.00	32.95	151.79	246.68	-9.74	0.0423	
4	RNASeq.37977	0.00	0.00	42.63	57.91	39.58	-9.67	0.0205	
5	RNASeq.5695	0.00	0.00	5.71	22.67	34.63	-9.64	0.0335	
6	RNASeq.47861	0.00	0.00	24.27	86.94	146.15	-9.63	0.0321	
7	RNASeq.18583	0.00	0.00	37.90	41.03	125.39	-9.42	0.0215	
8	Potrs006854g33925	0.00	0.00	29.96	86.88	48.55	-9.36	0.0205	
9	Potrs003850g05746	0.00	0.00	10.75	3.01	31.48	-9.33	0.0347	leucine zipper protein
10	RNASeq.44057	0.12	0.00	86.30	28.90	157.76	-9.32	0.0407	
11	Potrs003791g29788	0.00	0.00	3.57	1.59	5.28	-9.30	0.0206	Phosphomethylpyrimidine synthase, chloroplastic
12	Potrs007984g11196	0.00	0.00	7.02	7.41	29.68	-9.17	0.0231	Peptidyl-prolyl cis-trans
13	Potrs040177g25403	0.00	0.00	9.97	15.87	17.04	-9.03	0.0205	uncharacterized protein LOC105115473
14	Potrs006408g09027	0.00	0.00	25.59	25.82	28.22	-9.02	0.0205	Chaperone protein DnaJ
15	RNASeq.41747	0.00	0.00	37.07	47.26	50.81	-9.01	0.0205	
16	RNASeq.7567	0.00	0.00	15.24	51.49	51.02	-8.99	0.0216	
17	Potrs020009g22834	0.00	0.00	10.82	8.76	16.94	-8.92	0.0205	Ethylene-responsive transcription
18	RNASeq.46539	0.00	0.00	27.28	41.15	16.84	-8.87	0.0205	
19	Potrs003649g29217	0.00	0.00	6.68	2.81	2.58	-8.81	0.0205	Serine carboxypeptidase-like
20	Potrs018399g35871	0.00	0.00	58.21	61.64	40.16	-8.75	0.0205	conserved unknown protein
21	RNASeq.32308	0.00	0.00	17.54	27.64	27.53	-8.74	0.0205	
22	RNASeq.16418	0.00	0.00	1.66	17.69	19.69	-8.65	0.0450	
23	Potrs035791g35223	0.00	0.00	25.72	34.68	33.41	-8.51	0.0205	CLAVATA3/ESR (CLE)-related
24	Potrs006080g29018	0.00	0.00	4.69	16.17	12.69	-8.47	0.0212	uncharacterized protein LOC105114046
25	RNASeq.10673	0.17	0.00	21.62	83.12	33.01	-8.43	0.0321	
26	Potrs031907g32627	0.00	0.00	9.25	16.15	13.90	-8.41	0.0205	protein N-like
27	Potrs004143g06038	0.00	0.00	55.80	111.96	23.44	-8.39	0.0216	
28	Potrs010001g15129	0.00	0.00	8.17	14.93	5.21	-8.38	0.0206	Uncharacterized protein At4g04980
29	RNASeq.44603	0.00	0.00	7.17	36.39	21.20	-8.33	0.0231	
30	RNASeq.40672	0.00	0.00	4.71	12.60	44.76	-8.33	0.0300	

One gene of interest is the copper transport protein found to be upregulated in resistant genotypes. Annotation information for the top downregulated genes in RG is limited but includes a DnaJ chaperone protein, phosphomethylpyrimidine synthase (chloroplastic), and leucine zipper protein (Table 3).

## 4. Discussion

### 4.1. Effect of nickel toxicity to trembling aspen seedlings

No visible physical damage was observed in seedlings treated with the nitrate solution controls or with water suggesting that excess nitrate is not impacting plant health. Furthermore, the significant increase in plant damage seen at the highest nickel dose of 1,600 mg/kg is likely due to the excess of nickel and not nitrate because the corresponding nitrate control dose did not cause any significant damage.

The 1,600 mg/kg nickel treatment had the widest range of damage among the samples tested. There were two moderately susceptible, and seven susceptible plants while three of the genotypes were classified as resistant because they had a damage rating of 1 or 3 at the end of the experiment. This supports the hypothesis that high genetic variability exists within *P*. *tremuloides* trees from metal uncontaminated or contaminated areas [10, 13]. This type of variability was seen in a similar analysis of *Betula papyrifera* populations [7]. This species is widespread in Northern Ontario and can survive in metal contaminated areas in the GSR. Like *Populus tremuloides*, this species has been found to accumulate nickel at high concentrations in the aerial parts of the plant [7, 9]. It suggests that at this threshold dose of bioavailable nickel, plant phenotypes begin to segregate possibly due to genetic variation in metal tolerance ability. Epigenetic differences among the plant population may also exist which can affect gene expression in response to the heavy metal stress and there are varying levels of plant tolerance as a result.

### 4.2. Differentially Expressed Gene (DEG) analysis

Overall, there were more upregulated transcripts than downregulated transcripts in resistant genotype (RG). This is likely a reflection of overall decreased functioning of the susceptible plants as they are physically dying from the high dose of nickel treatment. This could result in less gene expression in susceptible genotype (SG) as indicated by the low number of upregulated transcripts in these samples (considered downregulated in RG).

The differences between resistant and susceptible genotypes were investigated using several methods. Gene set enrichment analysis (GSEA) found two different sets of GO terms that were enriched in either RG or SG. In resistant genotypes, the upregulated GO functions related to the kinesin complex, microtubule movement and cell-wall modification. Multiple studies associated microtubule and other cytoskeleton elements with abiotic stress [14]. It is suggested that these components may be involved in early signaling responses after exposure to a stressor like toxic concentration of heavy metals as seen in nickel-stressed and copper-stressed seagrass (*Cymodocea nodosa*) [15]. Recent findings with yeast cells that are resistant to diverse metals and oxidative stress showed that silver-resistant, iron-resistant, nickel-resistant, and oxidative stress-resistant yeasts had upregulated and mutated genes related to cell wall integrity and organization. Their cell wall integrity test results also revealed that they had increased cell wall integrity levels, indicating the importance of cell wall in stress resistance [16–18].

The activities associated with DNA integration, regulation of growth and negative regulation of programmed cell death were downregulated in resistant genotypes compared to susceptible samples. These fundamental processes may be upregulated in the susceptible plants because of the high amount of damage experienced due to the nickel treatment. Regulating growth and programmed cell death may be pathways through which the plant is attempting to cope with the heavy metal stress.

### 4.3. Pairwise comparison of resistant and susceptible trembling aspen genotypes

The biological process categories of carbohydrate metabolism, cellular protein modification and transport were highly differentially regulated between the two genotypes. These categories were among the highest percentages of associated genes for both upregulated and downregulated transcripts. This suggests that these processes are very dynamic in their part of the stress responses that are induced by nickel. Also, structural molecular activity was highly upregulated in resistant genotypes and almost no downregulated transcripts were detected. This may be related to the increased cell wall modification found in GSEA analysis for these samples. Modifications within the cell structures may be required for responding to the increase in absorbed nickel ions from the treated soil.

In terms of cellular location, ribosomal genes were more upregulated in nickel-resistant *P*. *tremuloides* when compared to susceptible samples. This is validated by the increase in translation gene activities for biological function of the DEGs seen in RGs. Interestingly, this similar trend was identified for nickel-resistant *B*. *papyrifera* genotypes but only when compared to the water control. When these RG were compared with nickel susceptible samples there was a downregulation of ribosomal activity and a decreased expression of binding and transporter genes [7].

### 4.4. Top differentially expressed genes from the pairwise comparison of RG vs SG

The annotated GO terms assigned to the top 50 differentially expressed genes (DEGs) were used to create GO category graphs. For biological function, multiple categories associated with stress response including response to stress, chemical, endogenous stimulus and external stimulus were upregulated. This validates that a stress response was induced in the poplar plants at this high dose of nickel.

Some highly upregulated genes of interest in RG from the Top 30 (Table 2) were characterized as a Dirigent protein 10, GATA transcription factor, Zinc finger protein, Auxin response factor, Bidirectional sugar transporter, thiamine thiazole synthase and copper transport protein.

One gene family found in the top differentially expressed genes are dirigent (DIR) proteins. These genes appear to be highly responsive to biotic and abiotic stresses in plants [19, 20]. The main function of DIR proteins is to mediate the production of lignans and lignins from monolignol plant phenols [21]. Under different types of abiotic and biotic stress, the lignin composition and content in plant tissues is also known to undergo changes [19]. In the case of heavy metals, one study comparing zinc-treated non-accumulating *Arabidopsis thaliana* and accumulating *Thlaspi caerulescens* plants found high expression of genes associated with biosynthesis of lignin in both. This expression was noticeably higher for the zinc hyperaccumulator species *T*.*caerulescens*, along with other related genes including dirigent proteins [22]. Despite having similar functions in general, there is variation in when and where dirigent proteins are expressed depending on the plant tissue and environmental conditions [20]. The specific protein found upregulated in this present study in resistant trembling aspen genotypes is dirigent protein 10. This protein appears to be mainly expressed in plant roots as seen in *Arabidopsis* [23] and water-stressed Chinese cabbage [20]. The high expression of this protein in nickel-resistant *P*. *tremuloides* roots tissue also corroborates this expression pattern. Furthermore, there is clear evidence of the major role of DIR10 in the formation of the Casparian strip (CS) in *Arabidosis* [24]. CSs are shaped like rings distributed throughout the plant root endodermis in vascular plants. These cell-wall modifications form a tight junction composed of lignin and they are an important part of many plant processes particularly selective water and nutrient uptake [24]. Notably, the DIR protein’s primary role in cell wall metabolism [25] may be related to the enrichment of genes associated with cell-wall modification found in the nickel-resistant trembling aspen genotypes in GSEA analysis.

A Zinc finger protein (ZFP) was also identified in the highly upregulated genes for RG. There are many types of ZFPs with varying functions but in general they act as transcription factors (TFs) and can modulate gene expression [26]. The Cys2/His-2-type (C2H2) ZFP evidently plays a large regulatory role in stress responses including oxidative stress in plants [27, 28]. However, in addition to C2H2 ZFPs, a variety of ZFPs could be involved in these pathways as seen in a comparative transcriptomics study of salt stressed-rice plants [28]. They found 10 upregulated and 12 downregulated zinc finger genes in saline-tolerant plants including the C2H2 and C3HC4 types [28].

A GATA transcription factor was identified, and it is another type of zinc finger protein [29]. Numerous GATA TFs have been cited in literature for abiotic stress tolerance in plants. One example was the observation that when *OsGATA16* is upregulated, it improves cold tolerance in rice [30]. Stress-responsive genes in plants are classified as either regulatory of functional genes [31]. Regulatory genes like the ZFP and GATA transcription factors seen in this study are often part of a network which regulates the gene expression of functional genes needed to produce the compounds that confer heavy metal stress tolerance [32].

Thiamine thiazole synthase is another enzyme that has been linked to abiotic stress. Its major role in thiamine biosynthesis and mitochondrial DNA damage protection is established but recent studies also suggest it may be upregulated in plants as part of the abiotic stress response [33]. This trend has been seen for different stressors including salinity, drought, flooding, and sugar deprivation [34–36]. These biosynthesis genes may be upregulated to produce more thiamine, an antioxidant which can mitigate the oxidative stress induced by most abiotic stressors [36, 37]. For example, an increase in total thiamine compounds was detected in maize seedling leaves under different stress treatments and this was most pronounced in the oxidative stress treatment [36]. Thus, the general role of thiamine as an antioxidant under stress conditions is also documented in Alkim *et al*. [38].

The bidirectional sugar transporter found in the top upregulated genes could be a component of the plant abiotic stress response via control of carbohydrate metabolism [39]. Yamada *et al*. [40] described the different roles of sugar transporters (ST) in the plant stress response and how they regulate the accumulation of sugars under various environmental stress conditions. A transcriptomic study of nickel-treated *Acer rubrum* also reported an upregulation of a sugar transporter in plants treated with a high nickel dose and suggested that it may be involved in carbon distribution [41].

An auxin response factor was among the highly upregulated genes in resistant genotypes. Auxin’s important role in various developmental processes is well known but it may also be involved in abiotic and biotic stress response signaling pathways [42]. Auxin could also play a role in heavy metal tolerance mechanisms like avoidance by inducing morphological changes in roots as suggested by Nkongolo *et al*. [8].

The Potrs038704g29436 transcript is the 26^th^ top DEG (Table 1) and it has been identified in *Populus tremuloides* and annotated as a coding gene for a metal ion binding protein that is involved in directing the movement of different metal ions. GO ontology predicted this transcript is a copper transport protein but it can also bind to other metals. Trembling aspen is a known accumulator species [9], so this transport protein may be upregulated in the nickel-resistant genotypes to transport and sequester the excess heavy metal. A comparison of nickel content in the roots and leaves of the resistant and susceptible plants could elucidate if excess nickel is being accumulated in the plant’s above-ground leaf tissues. Furthermore, RT-qPCR could be utilized to confirm the gene expression differences.

There was limited annotation information for the top 30 downregulated genes found in the resistant genotypes when compared to susceptible (Table 3). As a result, is it more difficult to infer about the gene expression dynamics that could be suppressed in resistant genotypes or upregulated in susceptible genotypes. Some genes of interest from this group include a probable carboxylesterase 13 (1. Potrs011578g16653), leucine zipper protein (9. Potrs003850g05746) and FKBP-type peptidyl-prolyl cis-trans isomerase (Potrs007984g11196).

Carboxylesterases are common enzymes found within plant cells and they are part of regular metabolic activities and the defense response [43]. The leucine zipper protein is in the same gene family as the Arabidopsis gene AT5G65310 which is involved in regulating abscisic acid mediated signaling. Abscisic acid (ABA) is a key signaling hormone that is important for modulating the plant’s defense pathways in response to variety of abiotic stressors including heavy metal stress [44]. Finally, the identified FKBP-type isomerase enzyme is related to the Arabidopsis gene AT3G25230 which been linked to some abiotic stressors like heat and cadmium.

The most current literature review considering both model and non-model plants identified different genes associated with nickel resistance that those detected in the present transcriptome analysis. These genes include 1-aminocyclopropane-1-carboxylic acid deaminase (*ACC*), high affinity nickel transporter family protein (*AT2G16800*), iron-regulated protein (*IREG*), glutathione reductase (*GR*), glutathione-s-transferase, Metal transporter (*NRAMP 1*,*2*,*3*,*4*), Nicotianamine synthase (*NAS3*), Putative transmembrane protein (*TMP*), Serine acetyltransferase (*SAT*), Thioredoxin family protein, Zn finger protein of *Arabidopsis thaliana* (*ZAT11*), and *MRP4* [7, 45–52].

It is known that metal stress can affect the expression and activity of important antioxidant enzymes needed to deal with Reactive Oxygen Species. For instance, though nickel does not directly generate ROS because it is a redox-inactive metal [2], it has been reported to stimulate antioxidant enzymes such as superoxide dismutase (*SOD*), catalase (*CAT*), ascorbate peroxidase (*APX*), and glutathione s-transferase [4–6] in plants. The activities of these genes appeared to be reduced based on a transcriptome analysis of white birch (*Betula papyrifera*). This result is possibly due to enzyme inactivation from direct binding of Ni^2+^ to a -SH group or histidine, Ni displacement of other metals in the active binding site, or indirectly [7].

Czajka *et al*. [53]. reported a significant repression of the *AT2G16800* gene in *Populus tremuloides* treated with 400 mg/kg, 800 mg/kg, and 1,600 mg/kg of nickel. Kalubi *et al*. [9] investigated how gene expression is affected by metal contamination in one Ni accumulator tree species, trembling aspen (*Populus tremuloides)* and one tree species red maple (*Acer rubrum*) that deals with excess Ni using the avoidance strategy. The transcript level was measured for target genes associated with nickel resistance including *NRAMP4*, *NAS3*, *AT2G16800* and *MRP4*. They found that *P*. *tremuloides* samples from both contaminated (Kelly Lake) and uncontaminated (St. Charles) areas in the Greater Sudbury Region had significantly higher gene expression than found in the *A*. *rubrum* samples. Expression of *AT2G16800*, and *MRP4* was significantly increased in the *P*. *tremuloides* samples from the contaminated site compared to the uncontaminated. *NAS3* had a high upregulation in *P*. *tremuloides* for both sites compared to *A*. *rubrum* samples. Finally, *NRAMP4* expression was also higher in *P*. *tremuloides* samples from both sites compared to *A*. *rubrum* but this was the smallest difference. Though the downregulation of the *AT2G16800* and *MRP4* genes in *A*. *rubrum* samples from a metal-contaminated site was apparent, the direct environmental factors could not be established yet. It is hypothesized that *P*. *tremuloides* may be more sensitive to the abiotic stressors in the contaminated site which may trigger the change in the gene expression of these stress related genes to tolerate the excess nickel.

A gene expression study on northern red oak (*Quercus rubra)* conducted by Djeukam *et al*. [54] found that other genes associated with metal-stress including ACC, SAT, and *NAS3* could be upregulated in response to high doses of nickel nitrate (800 mg/kg and 1,600 mg/kg). The red oak is another important species for the GSR because it is classified as a Ni/Zn accumulator and has the ability to store these metals in its leaves at high concentrations [55].

Effects of nickel ions on gene expression in conifer species has been recently studied. Boyd and Nkongolo [56] reported a significant increase of *AT2G16800*, and *NRAMP* expression in *Picea glauca* root samples treated with different concentrations of Ni compared to water. They also revealed that Ni induces an upregulation of *ACCD* at low and high doses tested in roots. Nickel treatments resulted in a downregulation of *GR* at low dose (150 mg/kg) in needles and an upregulation at low and high doses in roots [57].

Moarefi and Nkongolo [58] compared *Pinus strobus* and *P*. *banksiana* treated with different doses of nickel. They observed that soil nickel treatments induced a downregulation of high affinity- Ni transporter family *AT2G16800* and Natural resistance-associated macrophage proteins (*NRAMP3*) in both species with the severity increasing at high levels of Ni. A concentration-dependent response of 1-aminocyclopropane-1-carboxylic acid deaminase (*ACC deaminase*) in both species consistent with the hormetic effects was observed. Nickel triggered also a concentration-dependent downregulation of serine acetyltransferase (*SAT*) in *P*. *strobus* with the lowest concentration showing the highest repression. An opposite trend was observed in *P*. *banksiana* where Ni induced an upregulation of *SAT*.

Transcriptome analysis has also been used for studying the effects of nickel stress on ecologically important hard wood species found in the Greater Sudbury Region (GSR) in Canada. Theriault *et al*. [7] compared resistant and susceptible white birch phenotypes that were identified amongst a sample population treated with the same 1600 mg/kg nickel dose. Through RNA-seq analysis, significant differences in gene expression were detected amongst genotypes. Gene expression patterns were characterized including the downregulation of genes associated with ribosomal activities and translation in resistant genotypes. Furthermore, specific genes that were highly upregulated in nickel resistant birch genotypes compared to the susceptible samples were identified as good candidate genes for nickel resistance including two Nramp transporters, and Glutathione S-transferase [7].

Alternatively, the maple study by Nkongolo *et al*. [8] detected no significant differences among the RG and SG genotypes in the 1600 mg/kg nickel treatment group. However, the comparisons between the different nickel dose treatment groups and water controls did show significant differential gene expression. Particularly, this effect was seen when comparing the high nickel dose (1600 mg/kg) to the lower nickel dose or control (150 mg/kg and 0 mg/kg). For example, there was an upregulation of transcripts associated with transport and translation in the high dose group. It is apparent from this study that a change in gene expression in response to high heavy metal concentrations can be triggered at a threshold dose between 800 mg/kg and 1600 mg/kg [41]. The gene expression activities required to cope with the heavy metals are likely different from those in control plants or treated with a low concentration of 150 mg/kg corresponding to the bioavailable amounts of Ni at sites.

The gene expression data in previous studies showed that response to nickel is quite variable among species for a specific gene. This transcriptome analysis revealed that the spectrum of genes triggered by nickel toxicity is complex and their pathways likely intertwined. Surprisingly, none of the genes affected by Ni based on RT-qPCR analyses was detected in this transcriptome investigation, suggesting gene interactions in *P*. *tremuloides* in responses to Ni toxicity. Likewise, Li *et al*. [59] identified PtDIR and PeDIR genes that were differentially expressed in *Populus trichocarpa* in response to *Marssonina brunnea* and Phytohormones ((abscisic acid, salicylic acid, methyl jasmonate, and ethylene). The heatmap analysis in the present study revealed that none of these common genes was among the top 100 up or downregulated in *P*. *tremuloides* in response to Ni toxicity.

## 5. Conclusion

This study provided data to further characterize and add to the draft *Populus tremuloides* transcriptome. The high-throughput RNA-seq data generated 27–45 million paired-end reads per sample. After sequence alignment, two groups of transcripts consisting of 32,677 new isoforms and 17,349 novel transcripts respectively were added to the draft transcriptome. This improved sequencing data was used in the comparative transcriptomics analysis between nickel-resistant and nickel-susceptible *P*. *tremuloides* genotypes. Overall, 36,770 genes were considered expressed among the samples and included in DEG analysis. From this list, 2,890 genes were differentially regulated when resistant and susceptible genotypes were compared.

From a whole transcriptome level, an upregulation in ribosomal and translation activities was identified in the nickel-resistant plants. A gene from the top 50 differentially expressed group that encodes a metal binding transport protein was identified as a candidate gene for nickel resistance in trembling aspen. Other highly upregulated genes that are associated with many aspects of the abiotic stress response were identified in resistant genotypes including Dirigent protein 10, ZFP and GATA transcription factors, thiamine thiazole synthase, auxin response factor and a bidirectional sugar transporter.

Overall, next-gen RNA sequencing was a useful method to further elucidate the whole transcriptome in *Populus tremuloides* root tissues. It also enabled the comparison of resistant and susceptible heavy metal-stressed plants and identified new gene targets associated with the potential underlying nickel resistance and stress response mechanisms used by trembling aspen (*P*. *tremuloides*).

## Supporting information

S1 Fig Top enriched GO terms from GSEA for upregulated (positive) genes in nickel resistant genotypes when compared with nickel susceptible genotypes. (PDF)

S2 Fig Top enriched GO terms from GSEA for downregulated (negative) genes in nickel resistant genotypes when compared with nickel susceptible genotypes. (PDF)
