# Supplementary material for: Transcriptome analysis of trembling aspen (Populus tremuloides) under nickel stress
Source: PLoS One. 2022 Oct 13;17(10):e0274740. doi: 10.1371/journal.pone.0274740 (PMC9560071; doi:10.1371/journal.pone.0274740)
Supplement: S2 Fig — (PDF) [file pone.0274740.s002.pdf]

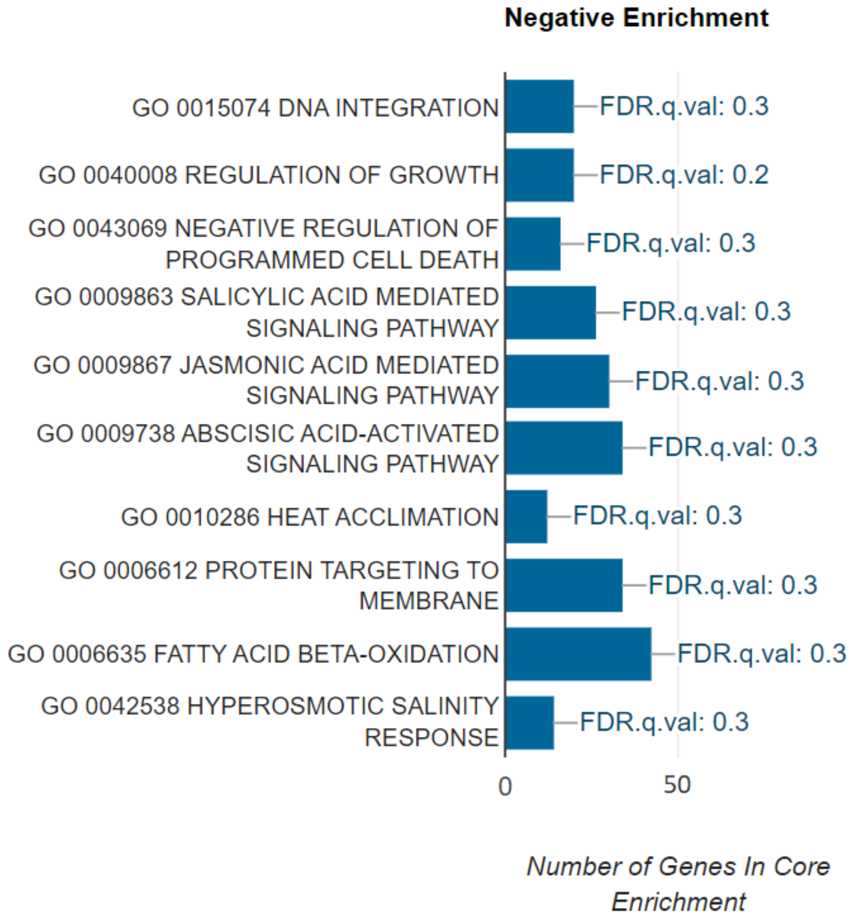

S2. Fig. Top enriched GO terms from GSEA for downregulated (negative) genes in nickel resistant genotypes when compared with nickel susceptible genotypes.
